# The Phosphatase Inhibitor Calyculin-A Impairs Clot Retraction, Platelet Activation, and Thrombin Generation

**DOI:** 10.1155/2017/9795271

**Published:** 2017-06-07

**Authors:** Renáta Hudák, János Vincze, László Csernoch, Ildikó Beke Debreceni, Tamás Oláh, Ferenc Erdődi, Kenneth J. Clemetson, János Kappelmayer

**Affiliations:** ^1^Department of Laboratory Medicine, Faculty of Medicine, University of Debrecen, 98 Nagyerdei krt., Debrecen 4032, Hungary; ^2^Department of Physiology, Faculty of Medicine, University of Debrecen, 98 Nagyerdei krt., Debrecen 4032, Hungary; ^3^Department of Medical Chemistry, Faculty of Medicine, University of Debrecen, 98 Nagyerdei krt., Debrecen 4032, Hungary; ^4^Department of Hematology, Inselspital, University of Bern, Murtenstrasse 40, 3008 Bern, Switzerland

## Abstract

The aim of this study was to investigate the effect of the serine/threonine protein phosphatase inhibitor, calyculin-A (CLA), on clot formation and on the procoagulant activity of human platelets. Platelet-rich plasma (PRP) samples were preincubated with buffer or CLA and subsequently platelets were activated by the protease-activated receptor 1 (PAR-1) activator, thrombin receptor activating peptide (TRAP). Clot retraction was detected by observing clot morphology up to 1 hour, phosphatidylserine- (PS-) expression was studied by flow cytometry, and thrombin generation was measured by a fluorimetric assay. For the intracellular Ca^2+^ assay, platelets were loaded with calcium-indicator dyes and the measurements were carried out using a ratiometric method with real-time confocal microscopy. CLA preincubation inhibited clot retraction, PS-expression, and thrombin formation. TRAP activation elicited Ca^2+^ response and PS-expression in a subset of platelets. The activated PRP displayed significantly faster and enhanced thrombin generation compared to nonactivated samples. CLA pretreatment abrogated PS-exposure and clot retraction also in TRAP-activated samples. As a consequence of the inhibitory effect on calcium elevation and PS-expression, CLA significantly downregulated thrombin generation in PRP. Our results show that CLA pretreatment may be a useful tool to investigate platelet activation mechanisms that contribute to clot formation and thrombin generation.

## 1. Introduction

Platelets play a crucial role in the pathogenesis of atherosclerotic diseases including acute coronary syndrome or ischemic stroke that are leading causes of death and disability worldwide. These events are triggered by disruption of the endothelium and plaque rupture or during interventions on coronaries, when platelets are tethered to surface-bound von Willebrand Factor (vWF), which initiates platelet activation and allows them to adhere to subendothelial components.

Protease-activated receptor 1 (PAR-1), the primary platelet thrombin receptor, is G-protein-coupled. The activator of this receptor is the strongest platelet agonist and an important contributor to atherothrombotic processes. Modulation of the PAR-1 receptor is the target for novel and promising antiplatelet drugs [1, 2]. PAR-1 receptor activation via thrombin or relevant thrombin receptor activating peptides (TRAPs) results in a series of signaling events terminating in platelet shape change and granule secretion via the G_12/13_ proteins and intracellular calcium release via the G_q_ mediated inositol triphosphate (IP_3_) pathway. Intracellular IP3 receptors can be directly activated by pharmacological agents like thiomersal that has been used previously as a calcium mobilizer and cell function-modulating agent [3].

Serine/threonine protein phosphatases (PP) are present in platelets predominantly as type 1 protein phosphatase (PP1) and type 2A protein phosphatase (PP2A) subtypes. Calyculin-A (CLA), a naturally occurring phosphatase inhibitor, present in marine sponges, in nanomolar concentration, primarily inhibits PP2A and indirectly PP1 [4, 5]. Among the other frequently used PP inhibitors tautomycetin selectively inhibits PP1, while okadaic acid at nanomolar concentrations preferentially suppresses the activity of PP2A [6–8]. Both PP1 and PP2A can be found in the membrane and cytosolic fractions of resting platelets [9].

A previous study from our group has described the effects of calyculin-A on TRAP-stimulated human platelets [8] and it was concluded that phosphatase inhibition prevents platelet-derived microparticle (MP) formation and degranulation in TRAP-activated platelets.

Surface exposure of phosphatidylserine (PS) is increased during platelet activation or apoptosis [10] and PS-expression is a key event in the control of blood coagulation, localizing prothrombin activation to the platelet plug, and regulating thrombin generation [11]. The initiation of early platelet activation events such as Ca^2+^-influx can also be affected by CLA, which blocks any further propagation of platelet reactivity [12].

The aim of this study was to explore the effects of CLA on resting and activated platelets and to simultaneously investigate clot formation, platelet activation, and thrombin generation and their modulation in platelet-rich plasma. PS-expression, intracellular calcium responses using a novel cytosolic Ca^2+^ level measurement, clot retraction, and thrombin generation were studied with or without TRAP activation. We show here, for the first time, that in platelet-rich plasma CLA blocked clot retraction and inhibited cytosolic Ca^2+^ elevation upstream of the IP_3_ receptor, abolished PS-expression, and subsequently inhibited thrombin generation. Thus, we suggest that this phosphatase inhibitor can be utilized in a wide variety of platelet functional assays for exploring biochemical pathways during thrombus formation.

## 2. Materials and Methods

### 2.1. Antibodies and Reagents

For flow cytometric analysis of PS-exposure we used FITC-labeled Annexin-V, Annexin-V binding buffer (10x concentrate), from Becton Dickinson (San Jose, CA), and a monoclonal anti-human CD41-PE antibody from Dako (Glostrup, Denmark). For platelet preparation either for flow cytometry or for the thrombin generation assay or intracellular Ca^2+^ measurements we used the following materials: paraformaldehyde (PFA), dimethylsulfoxide (DMSO), bovine serum albumin (BSA), HEPES, apyrase from potato, and Arg-Gly-Asp-Ser (RGDS) which were obtained from Sigma-Aldrich (St Louis, MO).

We used thrombin receptor activating peptide (TRAP) and thiomersal from Sigma-Aldrich (St Louis, MO) as platelet agonists. The protein phosphatase inhibitor, CLA, was from Calbiochem (San Diego, CA). HEPES buffer for flow cytometry and thrombin generation methods contained 150 mM NaCl and 25 mM HEPES, pH 7.4. HEPES-buffered saline for intracellular Ca^2+^ level measurements contained 145 mM NaCl, 10 mM HEPES, 10 mM D-glucose, and 5 mM KCl, pH 7.4, supplemented with 0.1% (w/v) BSA, 100 *μ*M RGDS, 200 *μ*M CaCl_2_, and 0.1 U/mL apyrase. For some experiments during the analysis of free calcium levels instead of CaCl_2_ the previously described HEPES buffer was supplemented with 500 *μ*M ethylene glycol-bis(2-aminoethylether)-N,N,N′,N′-tetraacetic acid (EGTA) from Sigma-Aldrich (St Louis, MO). We shall refer to this buffer hereafter as Ca^2+^-free buffer.

### 2.2. Preparation of Platelet-Rich Plasma

Whole blood was drawn from healthy volunteers with no medications for at least 2 weeks prior to the experiments. Blood sample collection from volunteers was approved by the Ethical Committee of the University of Debrecen. The DEOEC RKEB/IKEB 4318-2015 protocol grants permission to draw citrated blood samples from patients and controls both for analysing plasma and platelets. Blood was anticoagulated with 0.105 M sodium citrate. Platelet-rich plasma (PRP) was prepared from venous whole blood by centrifugation at 170 ×g for 15 minutes at room temperature (RT). Platelet count of PRP was adjusted to 250 G/L by adding platelet poor plasma (PPP). PPP was obtained by centrifugation of the citrated blood sample at 1500 ×g for 15 minutes at RT. In subsequent experiments the following PRP samples were analysed: (i) nonactivated (NA), (ii) CLA-pretreated nonactivated (NA + CLA), (iii) TRAP-activated (TRAP), and (iv) CLA-pretreated TRAP-activated (TRAP + CLA). In preliminary experiments 50 nM CLA was found to inhibit degranulation of platelets as well as PAC-1 binding without being toxic to cells so this concentration of CLA was applied in the experiments. TRAP was used at a final concentration of 20 *μ*M.

### 2.3. Clot Retraction Analysis

PRP (720 *μ*L) was preincubated with buffer control or CLA for 30 minutes at 37°C in a water bath and then activated by TRAP. In a glass tube, 800 *μ*L of every four samples was incubated with CaCl_2_ (at a final concentration of 25 mM) for 60 minutes at 37°C in a water bath. At time points 0, 20, 40, and 60 minutes, photos were taken to document clot formation. At the end of the experiment, the volume of the extruded serum was determined by an analytical scale. The amount of fibrin monomer in the extruded serum was measured by a latex enhanced quantitative immunoassay (Stago, Asniére, France) on the ACL-TOP coagulation analyser (Instrumentation Laboratory, Bedford, MA).

### 2.4. Flow Cytometric Assays

PRP (110 *μ*L) was preincubated with either HEPES buffer containing 0.5% DMSO as control or the protein phosphatase inhibitor CLA, for 30 minutes at 37°C in a water bath. CLA was used at a final concentration of 50 nM. After preincubation, platelets were activated either by TRAP at a final concentration of 20 *μ*M or by thiomersal at a final concentration of 200 *μ*M for 15 minutes at 37°C in a water bath. Then PRP (5 *μ*L) was stained with 5 *μ*L monoclonal CD41-PE antibody and 5 *μ*L Annexin-V-FITC and Annexin-V binding buffer (1x concentrate) was added to examine the PS-expression of the platelets. In each experiment 10,000 events were collected in the platelet gate, measured by an FC500 flow cytometer, and results were analysed with the Kaluza software (Beckman Coulter, CA).

### 2.5. Thrombin Generation Assay

Eighty microliters of pretreated PRP was incubated with 20 *μ*L of standard preparations of 1 pM recombinant tissue factor (rTF, PRP-Reagent, Thrombinoscope BV, Maastricht, The Netherlands) for 10 minutes in round-bottomed 96-well black microplates. For each PRP sample a simultaneously run calibrator, a stable complex of *α*_2_-macroglobulin (*α*_2_M) and thrombin (Calibrator-Reagent, Thrombinoscope BV, Maastricht, The Netherlands), was used to eliminate the differences between samples [13]. Thrombin generation was initiated by the addition of 20 *μ*L of a mixture of fluorogenic substrate and Fluo-Buffer that contained CaCl_2_ (Thrombinoscope BV, Maastricht, The Netherlands). Fluorescence was detected by a Fluoroskan Ascent® fluorimeter (Helsinki, Finland) and the thrombin generation curves were analysed by the Thrombinoscope software (Thrombinoscope BV, Maastricht, The Netherlands). Thrombin generation curves were characterised by the following parameters (calculated and presented by the Thrombinoscope software): Lagtime is the delay in minutes until thrombin formation starts. Peak thrombin expressed in nanomoles is the highest thrombin level and the time required to reach the peak thrombin level is designated as time to peak (in minutes). Velocity index (in nanomoles per minute) is the slope between lagtime and time to peak.

### 2.6. High-Speed Confocal Measurement of Platelet Cytosolic Ca^2+^ Levels

For measurements of intracellular Ca^2+^ levels, PRP was prepared as described above and RGDS peptide was added to prevent aggregation [14]. PRP (100 *μ*L) was loaded with 1.5 *μ*L of 2 mM Fluo-4-AM for 10 minutes at 37°C in a water bath. The cells were coloaded with Fura-Red by addition of 2 *μ*L of 1 mM Fura-Red-AM for 30 minutes at 37°C in a water bath. Before centrifugation, 2 mL of HEPES-buffered saline was added and after centrifugation at 350 ×g for 20 minutes at RT, the coloaded platelets were collected and resuspended in 100 *μ*L of the same buffer.

Platelets loaded with the calcium-indicator dyes were imaged using a Zeiss LSM 5 LIVE (Carl Zeiss AG, Jena, Germany) high-speed confocal scanning unit with a 40x oil immersion objective (NA: 1.3). The final 100 *μ*L solution was pipetted onto a glass coverslip secured above the objective within a temperature-controlled chamber and was allowed to rest for 2 minutes before the measurement. Both the chamber and the objective had been preheated to 37°C and this temperature was kept constant during the measurement. Frames (*x*-*y* images) were recorded at a rate of 5 Hz for 5 minutes using 488 nm excitation wavelength and two detection channels: a band pass filtered channel between 500 and 525 nm for the Fluo-4 signal and a long pass filtered channel above 635 nm for the Fura-Red signal. The wide gap between the cutoff wavelengths ensured that there was minimal crosstalk between the channels.

Details of the ratiometric method are demonstrated in Supplementary Figure  1 in Supplementary Material available online at https://doi.org/10.1155/2017/9795271. The green (Figures S1A, S1D, and S1G) and red (Figures S1B, S1E, and S1H) fluorescence channels were measured separately; then composite images were formed where color changes from red to green indicated increased intracellular Ca^2+^ concentrations (Figures S1C, S1F, and S1I). All platelets that could be observed for at least 30 s before and 90 s after the addition of TRAP were manually marked as regions of interest (ROIs) on the recorded image series. Time series curves of fluorescence values were analysed using a custom-built computer program. The average fluorescence level within a ROI on each channel was determined for all frames (*F*_Fluo-4_, green, and *F*_Fura-Red_, red; Figures S1J and S1M). Exponential fitting was used to determine the baseline fluorescence intensity on each channel (*F*_0(Fluo-4)_ and *F*_0(Fura-Red)_) taking into account the different bleaching levels for the two dyes during the experiment. Relative fluorescence values for the Fluo-4 (*F*′_Fluo-4_, green) and Fura-Red (*F*′_Fura-Red_, red) channels were calculated from the original fluorescence values by normalization to the respective baseline fluorescence curve (Figures S1K and S1N). Fluorescence ratio (*F*′_Fluo-4_/*F*′_Fura-Red_, blue) values corresponding to the intracellular Ca^2+^ level in the platelet were calculated by dividing the relative fluorescence value for the Fluo-4 channel by that of the Fura-Red channel at every time point (Figures S1L and S1O). Calcium transients, but not nonspecific changes of fluorescence intensity, are characterised by a simultaneous negative deflection of the *F*′_Fura-Red_ and positive deflection of the *F*′_Fluo-4_ curves and thus the *F*′_Fluo-4_/*F*′_Fura-Red_ trace. The magnitude of the transients can be characterised by the amplitude measured on the fluorescence ratio curve.

TRAP was added by pipetting 4 *μ*L of 500 *μ*M TRAP solution to the platelet suspension on the coverslip. Measurement was continuous during the addition of TRAP.

### 2.7. Statistical Analysis

Normality of the data was evaluated by the Kolmogorov-Smirnov test. Data are expressed as mean ± SD or mean ± SEM. Predetermined pairwise differences were analysed by paired Student's *t*-test and *p* values less than 0.05 were considered as statistically significant. In case the data showed a nonnormal distribution, the Wilcoxon signed rank-sum test was used for statistical evaluation. 95% confidence intervals for proportions were calculated using the modified Wald method.

## 3. Results

### 3.1. The Effect of CLA on Clot Retraction

**Figure 1:**
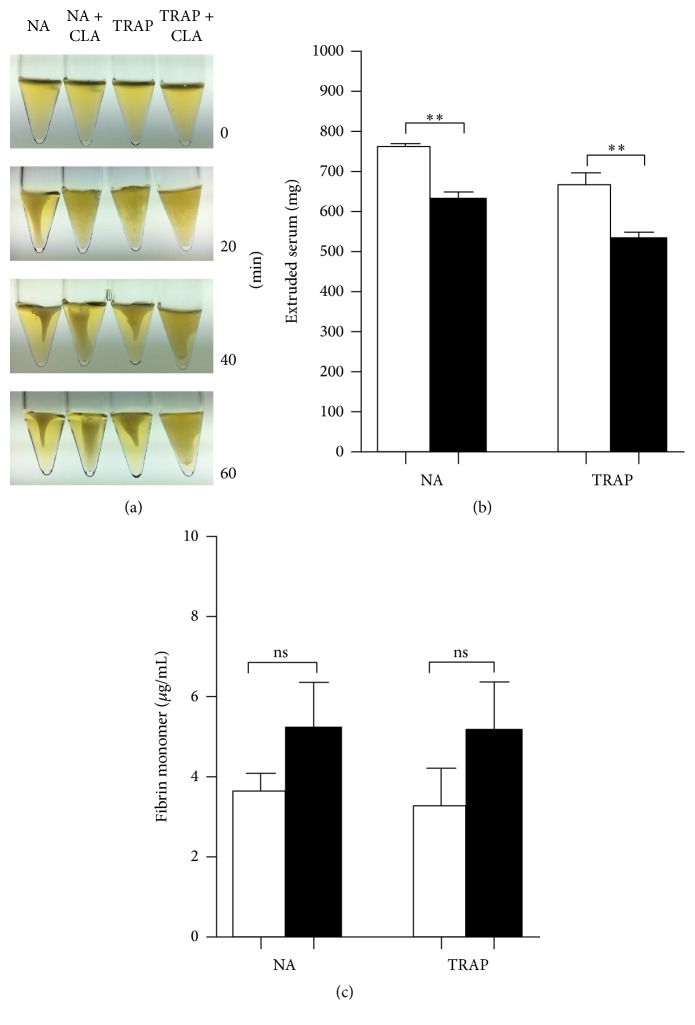
Inhibitory effect of CLA on clot retraction. Nonactivated (NA) and TRAP-activated samples displayed an intense clot retraction by 60 minutes, while CLA-pretreated samples were much less retracted (a). This significant difference could be observed by the lower quantity of extruded serum in CLA-pretreated samples (b) and a tendency could be observed to display higher fibrin monomer concentrations in CLA-pretreated samples (c). Nonactivated and activated samples are indicated with open bars and CLA-preincubated samples are represented by the black bars. The results are the mean and standard error of the mean (SEM) of 5 different experiments. Statistical significance was assessed by paired *t*-test. ^*∗∗*^*p* < 0.01. ns = nonsignificant.

It could be observed that clot formation and retraction commenced already by 20 minutes and by 60 minutes both the nonactivated and the TRAP-activated samples displayed an intense clot retraction. The CLA-pretreated samples however were much less retracted (Figure 1(a)). In accordance with this morphology the CLA-pretreated samples extruded significantly less serum (Figure 1(b)). The fibrinogen concentration was unmeasurably low in the samples by 60 minutes; nevertheless the fibrin monomers (FM) were determined by a quantitative assay and it was found that in the extruded sera of CLA-pretreated samples there was a tendency to higher FM values compared to nonpretreated samples (Figure 1(c)).

### 3.2. The Effect of CLA on the Level of Platelet PS-Expression

**Figure 2:**
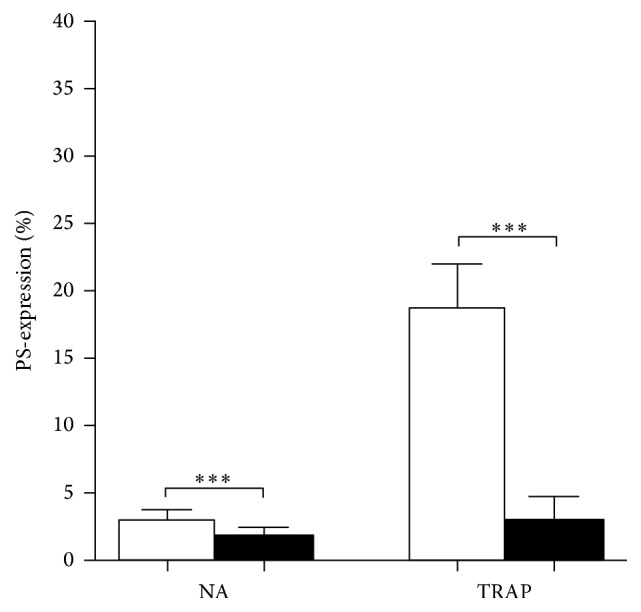
Inhibitory effect of CLA on PS-expression. Nonactivated (NA) and activated samples are indicated with open bars and CLA-pretreated samples with black bars. The results are the mean and standard deviation (SD) of 8 different experiments. Statistical significance was assessed by paired *t*-test. ^*∗∗∗*^*p* < 0.001.

In a series of experiments we determined the level of PS-exposure to analyse the effects of CLA on resting and activated platelets. PRP samples were preincubated with HEPES buffer (control) containing 0.5% DMSO or 50 nM CLA in DMSO for 30 minutes. Platelets were identified according to their CD41-PE staining; then PS-expression was determined by Annexin-V positivity. The PS-expression of nonactivated (NA) samples (Figure 2) was low (mean ± SD = 3.03 ± 0.84) and it was further decreased by CLA pretreatment (mean ± SD = 1.75 ± 0.67, *p* = 0.004). The PAR-1 receptor agonist, TRAP, significantly increased the number of platelets expressing PS (mean ± SD = 18.7 ± 3.23) compared to the NA sample (*p* < 0.0001) while CLA pretreatment completely prevented PS upregulation in TRAP-stimulated platelets (*p* < 0.0001) (Figure 2).

### 3.3. The Effect of CLA on Various Thrombin Generation Parameters

**Figure 3:**
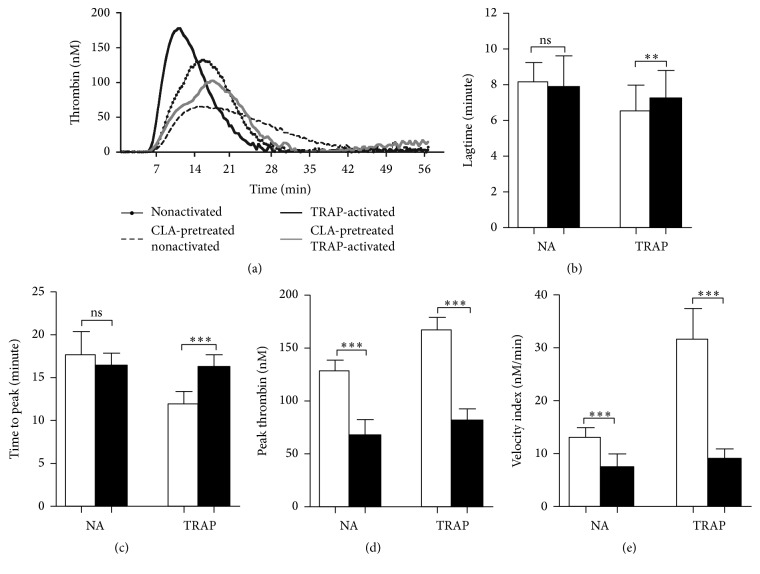
Thrombin generation assay and the effect of CLA on various parameters during thrombin generation. Thrombin generation in PRP was investigated as a procoagulant function of the intracellular Ca^2+^ level changes. The dotted line corresponds to nonactivated sample, the dashed line represents the CLA-pretreated nonactivated sample, black line marks the TRAP-activated sample, and CLA pretreatment on TRAP-activated sample is represented by the grey line (a). Lagtime (b) and time to peak (c) values were observed during TRAP-elicited platelet activation peak thrombin (d) and velocity index (e) values are informative about the amount and speed of generated thrombin. Nonactivated and activated samples are indicated with open bars and CLA-preincubated samples with black bars. The results are the mean and standard deviation (SD) of 5 different experiments. Statistical significance was assessed by paired *t*-test. ^*∗∗*^*p* < 0.01; ^*∗∗∗*^*p* < 0.001. ns = nonsignificant.

Representative thrombin generation curves demonstrate the effect of TRAP and/or CLA on thrombin formation by platelets (Figure 3(a)). The thrombin formation was faster, and the lagtime and time to peak were shorter in case of TRAP activation compared to the nonactivated sample (Figures 3(b) and 3(c)). TRAP activation increased the peak thrombin and also the velocity index (Figures 3(d) and 3(e)). CLA preincubation significantly prolonged the time of thrombin generation in TRAP-activated samples but did not have significant effect on the NA sample. CLA attenuated the peak thrombin and velocity index already in NA samples and completely blocked the TRAP-elicited augmentation in these parameters.

### 3.4. Ratiometric Measurement of Platelet Cytosolic Ca^2+^ Levels

Platelets marked on confocal time series images were divided into groups based on the time course and magnitude of calcium transients during the experiment.

**Figure 4:**
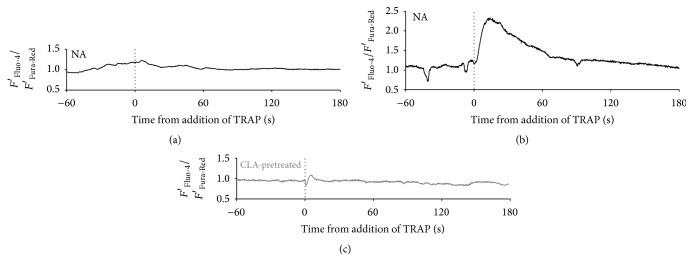
Characteristic changes of the cytosolic Ca^2+^ level of single platelets in relation to TRAP activation. Three typical time courses of the intracellular calcium concentration of platelets: no response to TRAP (a), transient increase in the Ca^2+^ level after the addition of TRAP (b), and no transient change upon CLA pretreatment (c). All curves are *F*′_Fluo-4_/*F*′_Fura-Red_ values where an increase indicates higher cytosolic calcium levels. Dotted lines mark the time of TRAP addition.

Platelet intracellular Ca^2+^ levels were measured after activation of nonactivated or CLA-pretreated sample. Two typical patterns of intracellular calcium concentration changes of platelets were observed on TRAP-activated samples without CLA pretreatment: no transient change after the addition of TRAP (Figure 4(a)) or a transient increase in the Ca^2+^ level (Figure 4(b)). When platelets were preincubated with CLA all calcium transients were abolished (Figure 4(c)). All curves depict *F*′_Fluo-4_/*F*′_Fura-Red_ values where an increase indicates higher cytosolic calcium levels. First, we investigated nonactivated platelets for 60 seconds and TRAP was added to the PRP during the microscopic measurement. Dotted vertical lines mark the time of TRAP addition. One hundred and seventeen platelets were investigated in TRAP-activated samples. In the case of samples that were not pretreated, 22.2% of platelets showed transient cytosolic Ca^2+^ increase upon TRAP addition. In case of platelets preincubated with calyculin-A, none of the investigated 122 cells showed calcium transients after TRAP administration during the time course of the measurement providing evidence for CLA blockage of Ca^2+^ elevation (Table 1). It is important to emphasize that CLA pretreatment did not affect resting cytosolic calcium levels (data not shown).

### 3.5. The Effect of Thiomersal on Platelet Activation

**Figure 5:**
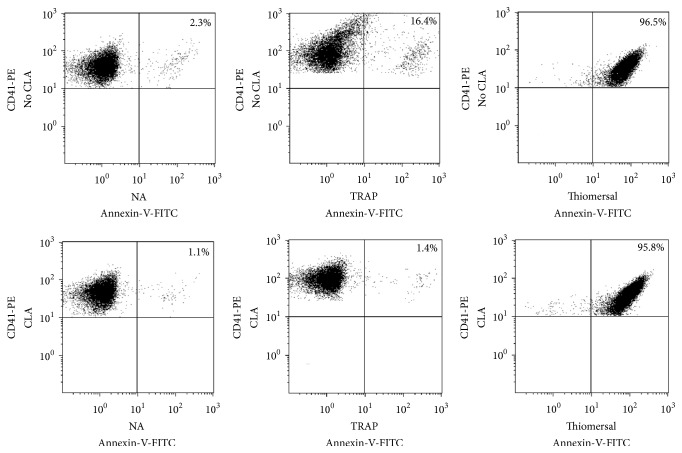
The effects of TRAP, thiomersal activation, and CLA preincubation on phosphatidylserine-expression (PS). The positivity of platelet PS-expression according to the Annexin-V labeling is in the upper right quadrants of the dot plots. The PS-expression of nonactivated sample (NA) was very low. When platelets were preincubated with CLA the PS-expression further decreased. Representative dot plots show that TRAP activation resulted in elevated PS-expression, which was completely blocked by preincubation with CLA. Thiomersal activated almost all of the platelets and this activation could not be inhibited by CLA preincubation.

Both nonactivated and CLA-preincubated samples were treated with thiomersal at a final concentration of 200 *μ*M and PS-expression was determined by flow cytometry. The PS-expression of nonactivated samples was 2.3% (Figure 5 (NA)) that was further decreased by CLA preincubation (Figure 5 (NA + CLA)). The ratio of PS-expressing cells was increased to 16.4% by TRAP activation (Figure 5 (TRAP)) that could be completely blocked by CLA. Contrary to the TRAP activation, thiomersal activation induced 96% PS-expression (Figure 5 (thiomersal)) that could not be prevented by CLA preincubation (Figure 5 (thiomersal + CLA)).

**Figure 6:**
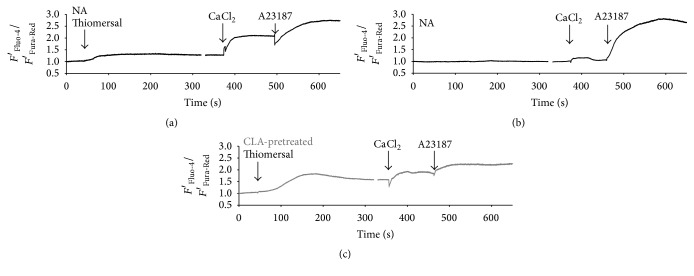
Changes of the cytosolic Ca^2+^ level of platelet groups in relation to activation by thiomersal in Ca^2+^-free buffer and the consequent store-operated calcium entry. Thiomersal evoked a prolonged elevation of cytosolic calcium in all the platelets followed by sustained store-operated calcium entry (a). Nonactivated platelets kept in calcium-free buffer show a minimal calcium entry in response to the resupplement of extracellular calcium by calcium chloride, probably due to platelets that got activated on physical contact to the glass coverslip (b). Thiomersal evoked a prolonged elevation of cytosolic calcium in CLA-pretreated platelets followed by sustained store-operated calcium entry, similarly to the response seen in NA platelets (c). All preparations responded to calcium ionophore A23187 with an increased calcium level. All curves are *F*′_Fluo-4_/*F*′_Fura-Red_ values where an increase indicates higher cytosolic calcium levels. Arrows mark the addition of thiomersal.

Thiomersal evoked a prolonged elevation of cytosolic calcium in all the platelets followed by sustained store-operated calcium entry (Figure 6(a)). Nonactivated platelets kept in calcium-free buffer show a minimal calcium entry in response to the resupplement of extracellular calcium by calcium chloride, probably due to platelets that were activated by physical contact with the glass coverslip (Figure 6(b)).

In CLA-pretreated platelets thiomersal also evoked a prolonged elevation of cytosolic calcium followed by sustained store-operated calcium entry, similarly to the response seen in NA platelets (Figure 6(c)). All preparations responded to calcium ionophore A23187 with increased calcium levels. All curves are based on *F*′_Fluo-4_/*F*′_Fura-Red_ values where an increase indicates higher cytosolic calcium levels.

## 4. Discussion

It is widely accepted that cell surface expressed, negatively charged phospholipids are required for the formation of multiprotein complexes of the coagulation cascade. Thus, this membrane dependency identified as PS-exposure can be regarded as a cellular control of coagulation processes during thrombus formation.

In our experiments we have investigated calcium signaling and PS-expression of platelets and investigated the role of phosphatase inhibition in these processes and subsequent thrombin formation in nonactivated and PAR-1 activated samples. Instead of thrombin we utilized TRAP, a potent activator of PAR-1 on platelets, to avoid early clotting and any potential pleiotropic effects [15].

Serine/threonine protein phosphatases play an essential role in cellular signaling, metabolism, and cell cycle control [7]. The activity of these phosphatases is needed for the initiation of platelet secretion and aggregation that is evident from the study which described that CLA suppressed aggregation, adhesion, secretion, and platelet spreading on fibrinogen [16].

Under experimental conditions in phosphatase-inhibitor-treated platelets, actin polymerization was inhibited, microtubules were reorganized in sustained pseudopods, and the phosphorylation of myosin light chain (MLC) was not enhanced upon thrombin stimulation [17]. The MLC phosphorylation is crucial in actomyosin contraction for platelet secretion, which is induced by MLC kinase and counterbalanced by myosin phosphatase including associated protein phosphatase-1 catalytic subunit and myosin binding (MBS/MYPT1) regulatory subunit [18].

When platelets are stimulated with thrombin, among several intracellular proteins, Rho-kinase, PKC, and integrin-linked kinase become activated resulting in the phosphorylation of MBS/MYPT1 (at Thr695/Thr696) causing a decreased myosin phosphatase activity accompanied with an elevated MLC phosphorylation [19, 20]. This leads to actomyosin contraction and subsequent secretion.

When CLA was added to platelets, PP was also blocked in this complex and this intervention further increased the phosphorylation of MYPT1 decreasing myosin phosphatase activity [8]. Already 10 nM CLA decreased phosphatase activity in both resting and TRAP-activated samples and with 50 nM the control phosphatase activity was decreased by more than 50%. Therefore, in the present study we used a final concentration of CLA of 50 nM.

Activation of platelets by various agonists is accompanied by a transient intracellular Ca^2+^ elevation, which is obligatory for the initiation and propagation of platelet responses such as adhesion, aggregation, and degranulation. The rise in intracellular calcium is a result of the calcium release from the intracellular stores mediated by the IP_3_ receptor. The depletion of the intracellular calcium stores is sensed by stromal interaction molecule (STIM1). Consequently, a calcium entry channel molecule (Orai1) is activated by STIM1 and elicits store-operated calcium entry (SOCE). This STIM-Orai regulated mechanism has been described in several cell types including platelets [21].

In earlier studies, it was found that CLA treatment of activated platelets suppressed the activation induced rise in Ca^2+^ levels measured by fluorescence spectrophotometric techniques [22, 23]. Experimental techniques have considerably improved in the past decades. More recently, a ratiometric flow cytometric method has been described that is suitable for measuring calcium fluxes in platelets [24]. We have investigated platelet cytosolic Ca^2+^ levels by a real-time ratiometric measurement with confocal microscopy, to more appropriately characterise changes in intracellular calcium signals. It was found that activating platelets by TRAP via the PAR-1 receptor causes a clear intracellular Ca^2+^ signal in a proportion of platelets that was abolished by CLA pretreatment and this effect was unrelated to resting calcium levels. In control samples, upon TRAP activation, a heterogeneous response was predictable [25, 26]. Indeed, we have observed two clear patterns: fast and robust Ca^2+^-transients in 22% of platelets and no Ca^2+^-response in the rest. Similar to the heterogeneity in calcium signals, not all platelets express PS upon activation. After platelet activation, these distinct platelet subpopulations may have different roles in the coagulation process, depending on their activation state and surface properties [27]. Similarly to calcium signals, not all platelets express PS upon activation; there is current evidence for platelet subpopulations, heterogeneity of platelet responses, and functions in the thrombus-forming process [28–30]. We attribute the two distinct Ca^2+^-response patterns to the existence of the above-mentioned platelet subpopulations. Response heterogeneity can be due to intrinsic differences between platelets in age and in receptor and signaling proteins. As a result, at least three subpopulations of platelets have been suggested in a thrombus: aggregating platelets with (reversible) integrin activation, procoagulant (coated) platelets exposing phosphatidylserine and binding coagulation factors, and contracting platelets with cell-cell contacts [28].

By using thiomersal, a direct activator of the intracellular IP3 receptors, we could verify both by flow cytometric PS-expression studies and by the ratiometric calcium measurement that PP inhibition by CLA does not impair direct IP3 receptor stimulation, so its effect is exerted upstream of the IP3 receptor.

The thrombin generation test was originally described for plasma [31] and has later been extended to PRP [32] and it was found that this test may reveal important interactions between platelets and the clotting system [33]. In our experiments the speed of thrombin generation as measured by the lagtime and time to peak parameters and the activity of generated thrombin as exemplified in the peak thrombin and velocity index parameters were shown to be the most useful parameters to evaluate the effect of CLA on the thrombin generation of PRP.

Raised Ca^2+^ levels lead to the exposure of PS on the platelet surface that serves as a site for the assembly of intrinsic and extrinsic tenase and prothrombinase complexes; thus it is critical for thrombin generation in PRP [34]. We have found that CLA on TRAP-activated platelets influenced platelet responses by eliminating calcium transients. It also abolished agonist-induced PS-exposure and consequently downregulated thrombin generation in PRP as demonstrated both by the speed of thrombin formation and by the amount of generated thrombin.

PS exposing platelets are procoagulant; however, they are nonadhesive and possess closed (or blocked) integrins; thus they are unlikely to participate in clot retraction. Nevertheless, in our experiments CLA also inhibited the platelet subpopulation that participates in clot retraction. Fibrinogen was clotted to insoluble fibrin so very little fibrin monomer remained in the extruded serum particularly in the TRAP-activated PRP. We have found that CLA attenuated thrombin formation that may have contributed to less crosslinked fibrin in the clot and thus more FM was detectable in the extruded serum.

Taken together, these findings indicate that inhibition of PP by CLA pretreatment can be regarded as a useful tool to investigate the platelet subsets that contribute to enhanced thrombin formation and clot retraction.

## 5. Conclusions

Calyculin-A effectively inhibits phosphatases in resting and activated platelets and can be used in a wide variety of platelet functional assays ranging from clot retraction, via calcium measurements, to thrombin generation. With the simultaneous use of selective platelet activators it can be utilized to dissect biochemical pathways during thrombus formation.

## Supplementary Material

Details of the ratiometric calcium measurement.

## Figures and Tables

**Table 1:** Proportion of platelets responding to activation by TRAP.

Sample	Total number of investigated platelets	Ca^2+^ transient in response to activation by TRAP		No transient change	
		*n*	%	*n*	%
TRAP-activated PRP	117	26	22.2% (15.59–30.62%)	91	77.8% (69.38–84.41%)
CLA-preincubated TRAP-activated PRP	122	0	(0.0–3.67%)	122	100% (96.33–100.0%)

Numbers in brackets indicate the 95% confidence interval; total number of samples = 13 for each dataset.
