# Supplementary material for: The Phosphatase Inhibitor Calyculin-A Impairs Clot Retraction, Platelet Activation, and Thrombin Generation
Source: Biomed Res Int. 2017 Jun 7;2017:9795271. doi: 10.1155/2017/9795271 (PMC5478853; doi:10.1155/2017/9795271)
Supplement: Supplementary file 1 — Details of the ratiometric calcium measurement. [file 9795271.f1.docx]

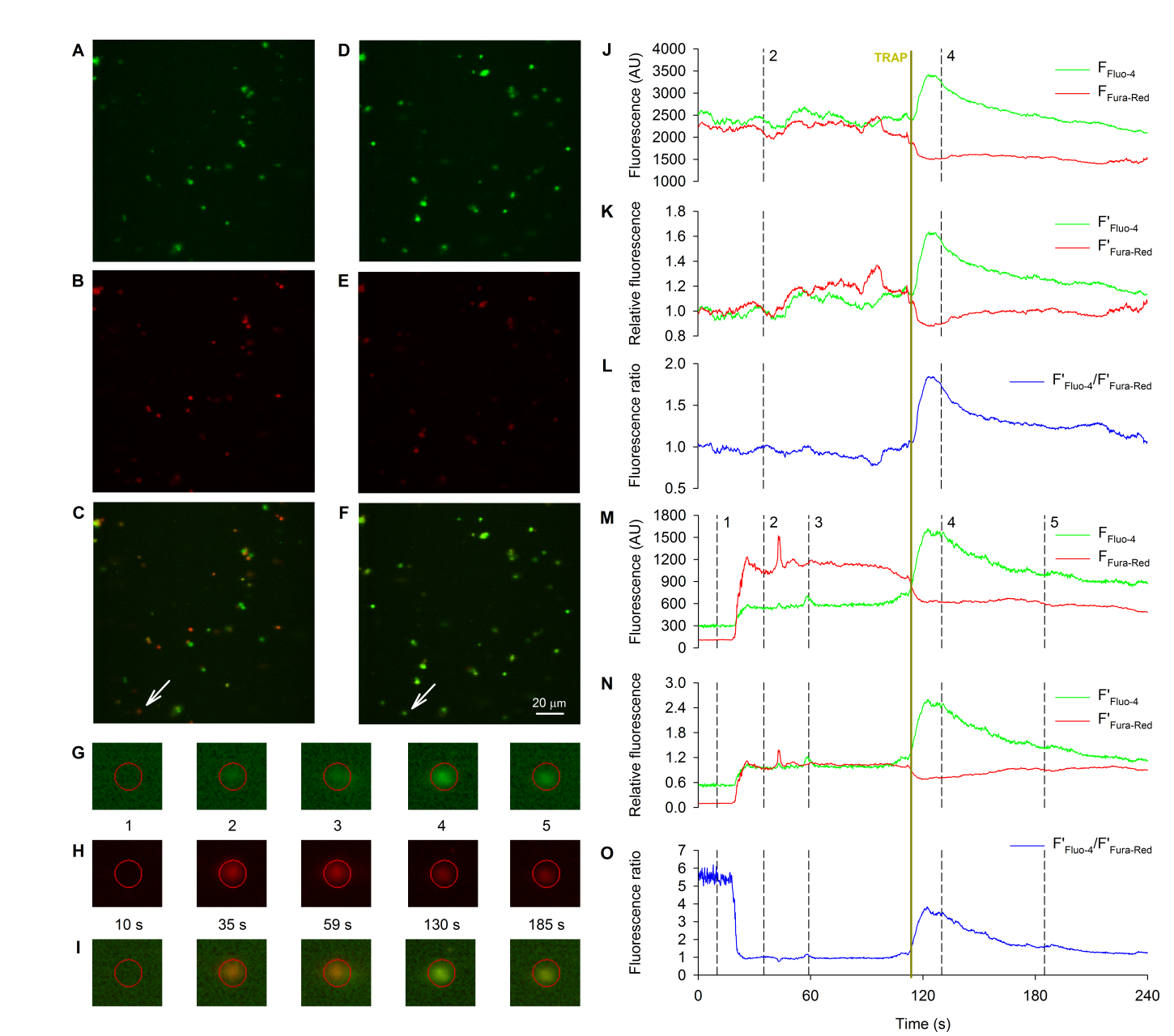


Supplementary figure 1. Ratiometric measurement of platelet intracellular Ca^2+^ levels by high-speed confocal microscopy. **A, B, C:** Fluo-4 (A), Fura-Red (B) and composite (C) fluorescence images showing the signal levels of non-activated platelets. **D, E, F:** Fluo-4 (D), Fura-Red (E) and composite (F) fluorescence images showing the signal levels of platelets 20 seconds after activation by TRAP. Panels A-F have the same scaling. Arrows on panels C and F point to the platelet shown on panels G, H and I. **G, H, I:** Excerpts of Fluo-4 (G), Fura-Red (H) and composite (I) fluorescence images showing the evolution of signal levels at the location of a stationary platelet. The red circle (diameter: 5.9 µm) denotes the ROI where the fluorescence levels were measured. **Excerpt 1:** the area before the arrival of any platelets with characteristic low fluorescence levels and high relative noise levels. **Excerpt 2:** baseline fluorescence of the platelet. **Excerpt 3:** only the fluorescence level of one of the channels is affected by another platelet floating into the ROI. **Excerpt 4:** a real calcium transient results in an increase of the Fluo-4 fluorescence and a decrease in the Fura-Red fluorescence. **Excerpt 5:** in the relaxation phase of the transient Fluo-4 fluorescence decreases while Fura-Red fluorescence increases relatively to its corrected background level.**J:** Original fluorescence values measured on all non-background pixels of the whole field of view on the Fluo-4 (F_Fluo-4_, green) and Fura-Red (F_Fura-Red_, red) channels. **K:** Relative fluorescence values for the Fluo-4 (F’_Fluo-4_, green) and Fura-Red (F’_Fura-Red_, red) channels calculated from the original fluorescence values measured on all non-background pixels of the whole field of view by normalization to the corrected background fluorescence level. **L:** Fluorescence ratio (F’_Fluo-4_/F’_Fura-Red_, blue) values corresponding to the intracellular Ca^2+^ level of all platelets in the field of view are calculated by dividing the relative fluorescence value for the Fluo-4 channel by that of the Fura-Red channel at every time point. Dashed lines on panels J, K and L correspond to panels A, B and C (2) and D, E and F (4). **M:** Original fluorescence values measured in the selected ROI on the Fluo-4 (F_Fluo-4_, green) and Fura-Red (F_Fura-Red_, red) channels. **N:** Relative fluorescence values for the Fluo-4 (F’_Fluo-4_, green) and Fura-Red (F’_Fura-Red_, red) channels calculated from the original fluorescence values by normalization to the corrected background fluorescence level. **O:** Fluorescence ratio (F’_Fluo-4_/F’_Fura-Red_, blue) values corresponding to the intracellular Ca^2+^ level in the platelet are calculated by dividing the relative fluorescence value for the Fluo-4 channel by that of the Fura-Red channel at every time point. Dashed lines on panels M, N and O correspond to images on panels G, H and I. The solid yellow line across panels J-O corresponds to the activation of platelets by TRAP.
